# Tricuspid transcatheter edge-to-edge repair in a 72-year-old patient with a left ventricular assist device and prior mitral edge-to-edge repair: a case report

**DOI:** 10.1093/ehjcr/ytae074

**Published:** 2024-02-21

**Authors:** Stephan Staubach, Michael Sailer, Johannes Koch, Anatol Maier, Andreas Jeron

**Affiliations:** Department of Cardiology, Rems-Murr-Klinikum Winnenden, Am Jakobsweg 1, 71364 Winnenden, Germany; Department of Cardiology, Rems-Murr-Klinikum Winnenden, Am Jakobsweg 1, 71364 Winnenden, Germany; Department of Cardiology, Rems-Murr-Klinikum Winnenden, Am Jakobsweg 1, 71364 Winnenden, Germany; Department of Cardiology, Rems-Murr-Klinikum Winnenden, Am Jakobsweg 1, 71364 Winnenden, Germany; Department of Cardiology, Rems-Murr-Klinikum Winnenden, Am Jakobsweg 1, 71364 Winnenden, Germany

**Keywords:** Left ventricular assist device (LVAD), Tricuspid regurgitation, Transcatheter edge-to-edge repair, Heart failure, Case report

## Abstract

**Background:**

We report a case of a 72-year-old patient developing a significant tricuspid regurgitation (TR) 6 years after a left ventricular assist device (LVAD) implantation. The aim of this case is to demonstrate the feasibility of transcatheter edge-to-edge repair (TEER) of the tricuspid valve and the excellent clinical benefit in long-term follow-up in an LVAD patient.

**Case summary:**

Our patient presented with recurrent acute heart failure syndrome. After a fulminant myocarditis in 2005, his previous treatment consisted of cardiac resynchronization therapy, TEER of the mitral valve, and LVAD (HeartMate III) implantation. At that point in time, his TR was only mild to moderate. Due to recurrent hospitalization despite optimized heart failure medication, we decided to treat the patient with a tricuspid TEER. His immediate post-interventional result and 1-year follow-up showed an excellent outcome with only minimal TR on transoesophageal echocardiogram.

**Discussion:**

In general, TR improves after LVAD implantation. However, there are two possible pathophysiological mechanisms, which result in an increasing TR: firstly, supporting LV dysfunction may lead to a leftward shift of the interventricular septum with restriction of the tricuspid leaflets. Secondly, the increase of venous preload with LVAD support may result in an annular dilatation with secondary TR, particularly in patients with pre-existing right ventricular dysfunction. According to the data currently available, the unpredictable course of developing TR necessitates regular clinical examination and echocardiographic investigation. Treatment with TEER appears to be feasible and safe, with excellent 1-year results in patients with previously implanted LVADs.

**Learning points**

- A dedicated echocardiographic transthoracic and occasionally transoesophageal examination is crucial to identify valve diseases, especially in patients with prior cardiac device implantations.
- A relevant tricuspid regurgitation can occur years later after left ventricular assist device (LVAD) implantation. Treatment with transcatheter edge-to-edge repair appears to be feasible and safe, with excellent long-term results in a patient with previously implanted LVAD.

## Introduction

Patients requiring long-term mechanical support with left ventricular assist devices (LVADs) may develop significant tricuspid regurgitation (TR) as a complication. There are two possible pathophysiological mechanisms: Supporting LV dysfunction can result in a leftward shift of the interventricular septum, leading to restriction or tethering of the tricuspid leaflets, especially in the presence of an enlarged right ventricle.^1^ The other mechanism leading to clinically relevant TR involves the increase of venous preload with LVAD support, particularly in patients with pre-existing impaired right ventricular (RV) function and annular dilatation.^2^ Therefore, the incomplete coaptation of the tricuspid valve leaflets contributes to progressive RV failure with volume overload, necessitating recurrent readmissions to hospital. Though tricuspid valve repair remains controversial in this setting, we present the technical feasibility of a tricuspid transcatheter edge-to-edge repair (TEER) in an LVAD patient and the importance of dedicated echocardiographic investigations.

## Summary figure

**Table:** 

Timeline of relevant events	
February 2005	Fulminant myocarditis leading to reduced ejection fraction. Coronary artery disease was excluded.
February 2016	Cardiac resynchronization therapy (CRT-D) for progressive dyspnoea.
April 2016	Transcatheter edge-to-edge repair of the mitral valve for severe regurgitation. Tricuspid regurgitation only mild to moderate at this stage.
July 2016	Left ventricular assist device (HeartMate III) implantation after cardiac arrest as a destination therapy.
2022	Repeated hospitalizations due to exacerbation of heart failure.
August 2022	Transoesophageal echocardiogram (TOE) shows torrential (V°) TR, which was treated with a TEER after heart team discussion.
August 2023	In 1-year follow-up, TOE demonstrates only minimal TR. Patient is in good clinical condition. No further need for readmission since the transcatheter repair in August 2022.

## Case presentation

**Figure 1:**
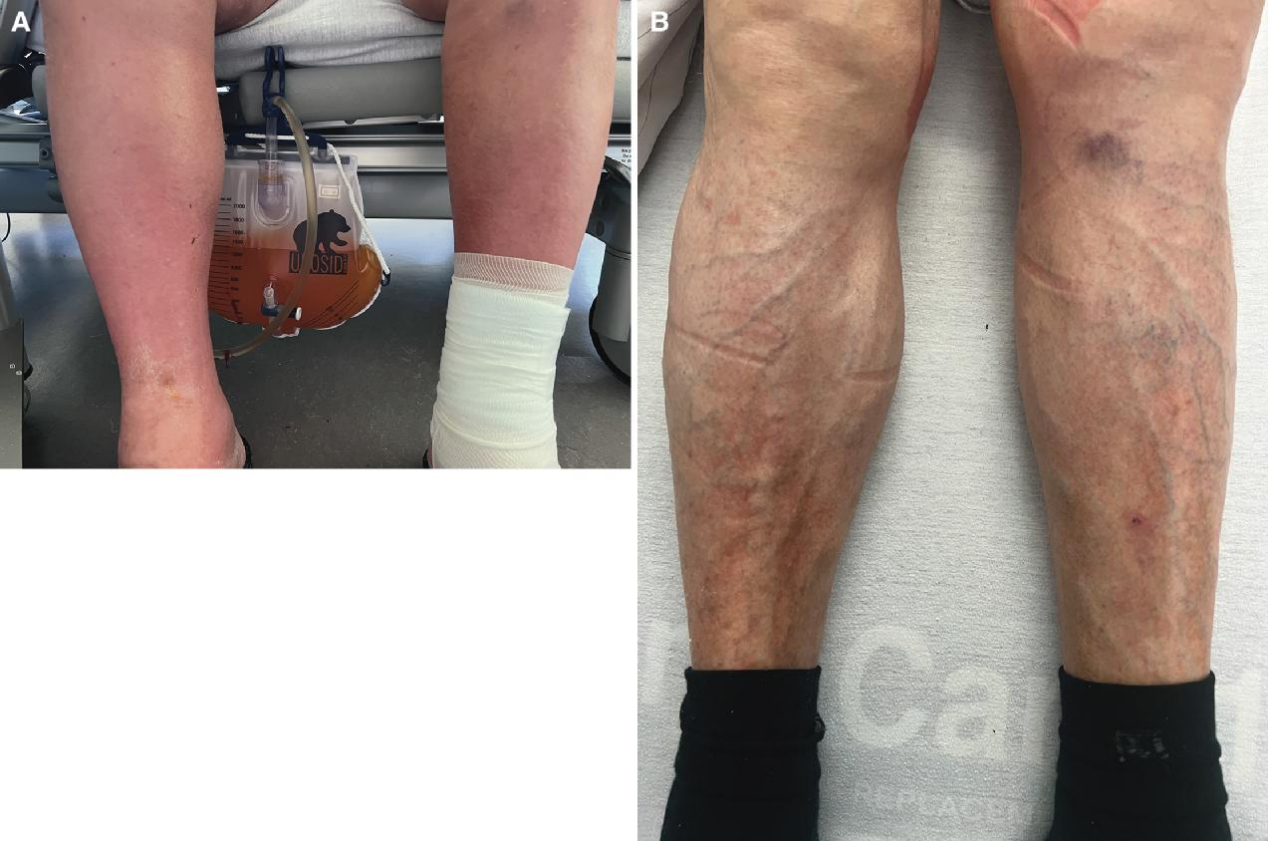
Leg oedema before (*A*) and 1 year after (*B*) successful edge-to-edge repair of torrential tricuspid regurgitation.

**Figure 2:**
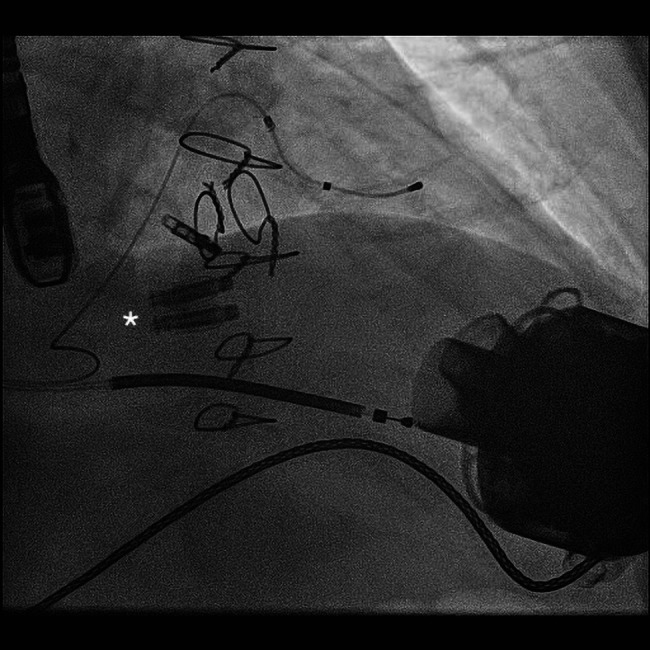
Chest X-ray illustrating left ventricular assist device, left ventricular and right ventricular leads, one MitraClip® system (Abbott Structural Heart, Santa Clara, CA, USA) in the mitral position, and two Pascal Ace® (Edwards Lifesciences, Irvine, CA; asterisk) in the tricuspid position.

A 72-year-old male patient presented with acute heart failure syndrome (AHFS), characterized by severe leg oedema (*Figure 1*), ascites, and moderate bilateral pleural effusions. He had been treated for fulminant myocarditis in 2005, which resulted in a reduced LV ejection fraction. Previous treatment consisted of CRT-D and anticoagulation due to persistent atrial fibrillation, TEER of the mitral valve, and LVAD (HeartMate III) implantation (*Figure 2*). At that point in time, his TR was only mild to moderate. A heart transplantation was refused by the patient.

**Figure 3:**
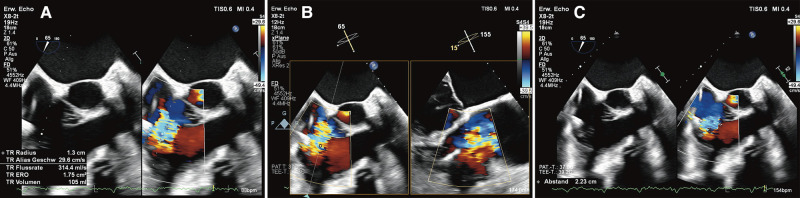
Tricuspid regurgitation with an effective regurgitant orifice area of 1.75 cm^2^ (*A*) and large regurgitation jet (*B*) with a vena contracta of 22 mm (*C*).

In the last months before TEER, he was hospitalized several times with symptoms of AHFS. His heart failure medication included furosemide (80 mg), bisoprolol (5 mg), and empagliflozin (10 mg). A mineralocorticoid receptor antagonist and an angiotensin receptor blocker were ceased due to low blood pressure and progressive renal dysfunction. The transthoracic echo showed a reduced LV ejection fraction of 25%. The right ventricle was severely dilated with slightly impaired RV function; however, the atrioventricular valves and the right heart could not be sufficiently visualized transthoracically. Due to image shadowing of the LVAD and CRT leads, a TOE was performed, which showed a torrential TR [vena contracta 22 mm, effective regurgitant orifice area (EROA) 1.75 cm^2^] caused by annular dilatation secondary to left-sided heart failure (*Figure 3* and *Video 1*; see Supplementary material online, *Videos S1* and *S2*) without any signs of lead-induced TR.

**Figure 4:**
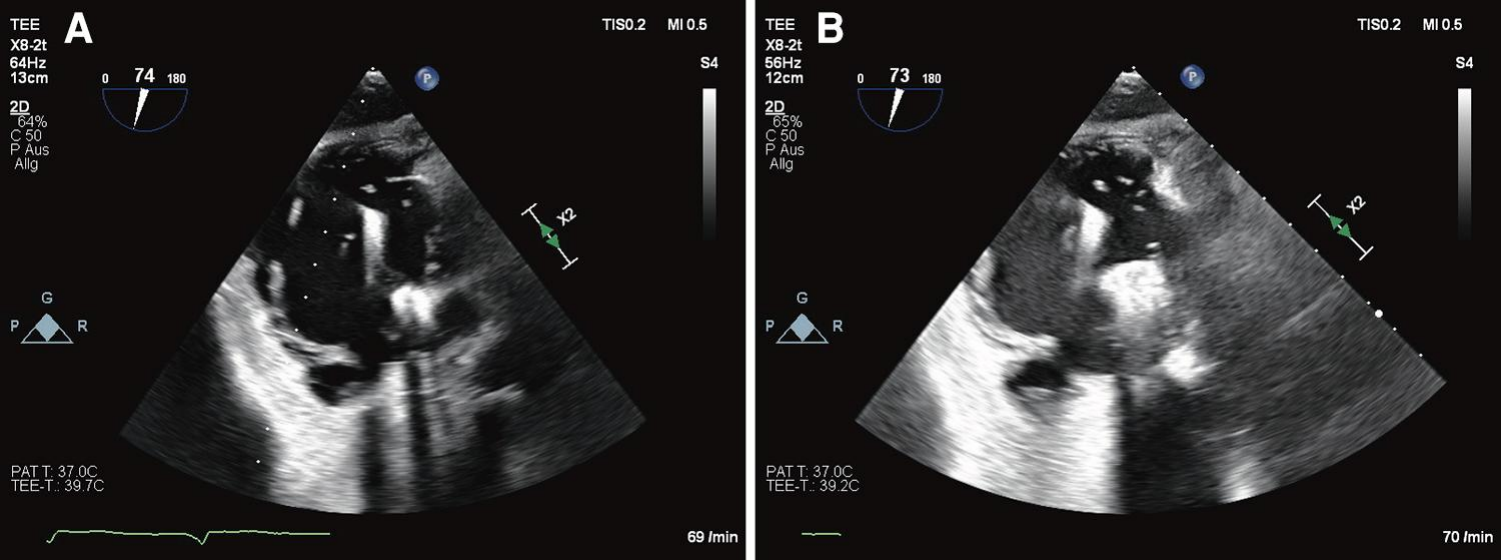
Procedural transoesophageal echocardiography showing the first implanted (*A*) and the second device (*B*) both in anteroseptal position in a transgastric short-axis view.

**Figure 5:**
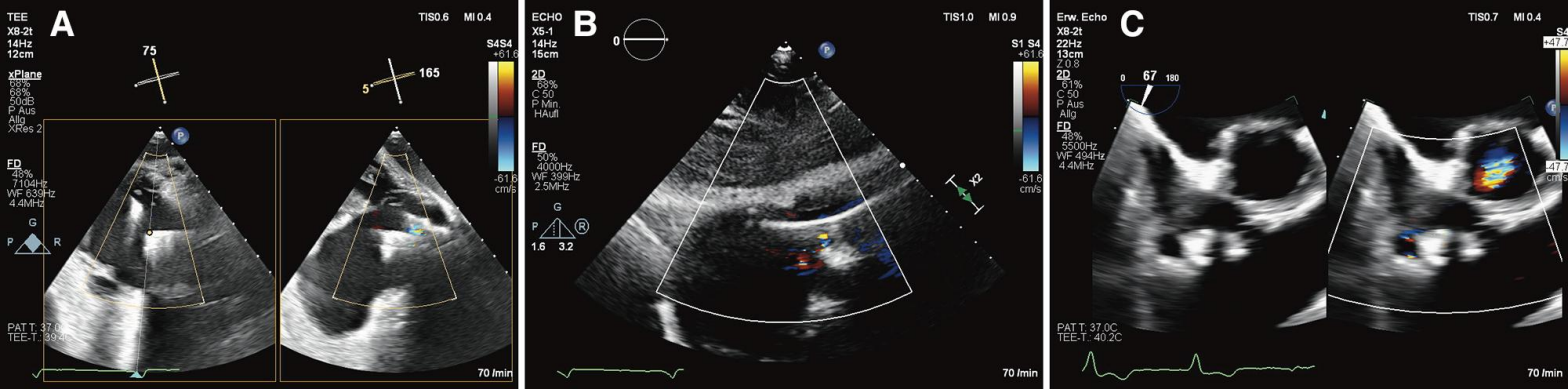
Immediate procedural result showing a minimal TR between Pascal Ace® (Edwards Lifesciences, Irvine, CA) and right ventricular lead (*A*). After 1 month, a stable result with only mild TR in subcostal view (*B*) is documented and in the inflow–outflow view (transoesophageal echocardiogram) at 1-year follow-up (*C*).

After a thorough literature search and internal discussion within the heart team, we decided to treat the patient with a TEER. This decision was based on a markedly elevated EuroSCORE II of 74.36% and TRI-SCORE of 48%. Treatment with a continuous intravenous furosemide infusion for 7 days achieved a deficit of 12 kg (*Figure 1*), which also resulted in a reduction of the gap between the anterior and septal leaflet (see Supplementary material online, *Video S3*) and TR grade (III–IV°). Right heart catheterization prior to the mitral TEER revealed a combined moderate pre- and post-capillary hypertension (pulmonary artery pressure 58/20/40 mmHg, pulmonary capillary wedge pressure 17 mmHg, and 4.9 wood units) and was not repeated ahead of the tricuspid TEER. Tricuspid regurgitation was successfully managed with two Pascal Ace® devices (Edwards Lifesciences, Irvine, CA) in anteroseptal position under general anaesthesia. Post-procedural echo revealed only a minimal remaining TR (*Figure 4* and *Video 2*). After discharging, the patient progressed well without further need for rehospitalization or relevant symptoms of right heart failure. His weight and diuretic dose remained stable for at least 18 months after the TEER procedure. Diuretic therapy was reduced to furosemide 40 mg, and valsartan 80 mg was added. The dose of phenprocoumon was able to be reduced significantly with less fluctuations in international normalised ratio measurements. His 1-year follow-up showed an excellent outcome with only minimal TR on TOE (*Figure 5* and *Video 3*). Figures demonstrate the patient’s severely oedematous legs prior to the TEER procedure and after 1 year during a TOE follow-up (*Figure 1*).

## Discussion

We present a case of a patient with a long and complex cardiac history, resulting in an end-stage congestive heart failure that warranted the implantation of an LVAD, CRT-D and mitral TEER. Subsequently, a tricuspid TEER also became necessary. To the best of our knowledge, this is the first case managed with a tricuspid valve TEER with the Pascal System® (Edwards Lifesciences, Irvine, CA) on the background of a prior LVAD implantation.

Apart from TEER, there are other CE-marked devices available for interventional treatment of TR. Examples include transcatheter annuloplasty or heterotopic valve implantation.^3^ A recent retrospective study, which included 122 patients, compared annuloplasty with TEER. The study found no significant difference in terms of technical success and improvement in TR severity.^4^ Another recent retrospective study comparing the two procedures showed an improved reduction of TR grade in patients treated with annuloplasty. However, this was associated with more clinically relevant haemorrhagic events.^5^ Randomized control trials comparing different TR procedures are still lacking.

We favoured the TEER procedure in this particular patient, due to a predominantly anteroseptal TR in a type I valve anatomy (three-leaflet configuration), a decreasing coaptation gap after extensive diuretic therapy, and limited echocardiographic view for an annuloplasty procedure. Had the screening results for the TEER procedure been unfavourable, a heterotopic valve implantation would have been a viable backup option, as well as in the case of a failed TEER procedure with insufficient reduction of TR.^6^

Our literature search found only one case of a 59-year-old female patient with a previously implanted LVAD and tricuspid valve repair as a bridge to transplantation.^7^ Due to recurrent TR, she was additionally treated with TEER with the MitraClip® XTR system (Abbott Structural Heart, Santa Clara, CA, USA), which reduced the EROA by 50% to 0.7 cm^2^. Thirty days after the TEER procedure, heart transplantation was performed on her.

Previous data have shown that TR becomes relevant in about one- to two-thirds of patients post-LVAD implantation.^8^ In our patient, the TR was mild to moderate during the LVAD implantation. In general, TR improves immediately after LVAD implantation. The reason for this is that LVAD support decreases end-diastolic LV pressures and subsequently also pulmonary pressures, which in turn leads to reduced RV afterload resulting in less functional TR. However, in some cases, TR becomes significant on LVAD support. This is thought to be due to increased preload as a result of higher venous return, which subsequently results in movement of the interventricular septum towards the left ventricle, leading to functional restriction of the tricuspid leaflets.^1^ Worsening TR is more likely to occur with pre-existing impaired RV function. This mechanism could be assumed in our patient. Furthermore, progression of pulmonary hypertension may also coincide with progression of TR. Current data indicate benefits to treating severe TR at an earlier disease stage, which can be assessed by the TRI-SCORE.^9^

A further study on patients captured in the European Registry for Patients with Mechanical Circulatory Support (EUROMACS) investigated the clinical impact and progression of uncorrected TR with LVAD implantation.^10^ In 2411 patients, an uncorrected TR pre- and post-LVAD was associated with increased early and late mortality (*P* = 0.015). The results indicated that over an observational period of 3 years, there was an improvement of TR without intervention, mainly driven by a decrease in pulmonary pressure and subsequent less or no dilatation of the right ventricle. Our patient, however, developed torrential TR with clinical sings of right heart failure years after the LVAD implantation. Therefore, structured and precise transthoracic and transoesophageal echocardiography is paramount before LVAD implantation to determine the aetiology of TR and assess the RV function. Measurements should include 3D RV function to screen for underlying myocardial disease of the left ventricle. Based on these findings, the decision should be made whether to perform concomitant valve surgery during LVAD implantation.

The International Society for Heart and Lung Transplantation (ISHLT) guidelines^11^ recommend consideration of a simultaneous surgical repair of the tricuspid valve during the LVAD implantation for moderate or severe TR (IIaC). However, a large series, which included six observational studies with 3249 patients, compared LVAD alone and LVAD plus tricuspid valve surgery. No significant differences emerged in terms of mortality or the requirement for RV assist devices.^12^ Recent data showed comparable results in terms of RV failure.^13^ Other authors suggest a more conservative approach to TR due to an observed decrease in regurgitation volume after LVAD Implantation.^10^ There is a paucity of data with regard to transcatheter interventions to the atrioventricular valves valves after mechanical circulatory support with a LVAD.

According to the data currently available, the unpredictable course of developing TR necessitates regular clinical examination and echocardiographic investigation to detect relevant and treatable TR.

## Supplementary Material

ytae074_Supplementary_Data

## Data Availability

The data underlying this article will be shared upon reasonable request to the corresponding author.

## Lead author biography

Dr Stephan Staubach is part of the structural heart team at the Department of Cardiology in Winnenden, Germany. He is specialized in transcatheter edge-to-edge repair of mitral and tricuspid valve. He is both an imager and interventional cardiologist.
